# Accuracy and precision of chronic myocardial infarct characterization with native T_1_ mapping at 3T

**DOI:** 10.1186/1532-429X-17-S1-P166

**Published:** 2015-02-03

**Authors:** Avinash Kali, Ivan Cokic, Hsin-Jung Yang, Behzad Sharif, Rohan Dharmakumar

**Affiliations:** Cedars-Sinai Medical Center, Los Angeles, CA USA

## Background

Native T1-maping at 3T has been shown to reliably characterize chronic myocardial infarctions (MIs). In this study, we evaluated the accuracy and precision of different thresholding techniques and visual delineation for characterizing chronic MIs on native T_1_ maps at 3T.

## Methods

Canines (n=23) underwent CMR at 4 months following MI. Native T1 maps (MOLLI; 8 TIs with 2 inversion blocks of 3+5 images; minimum TI=110ms; ΔTI=80ms; TR/TE=2.2/1.1ms) and Late Gadolinium Enhancement images (LGE; IR-prepared FLASH; TI optimized to null remote myocardium; TR/TE=3.5/1.75ms) were acquired at 3T. Infarct size and transmurality measured using Mean + 2 standard deviations (SD), Mean+3SD, Mean+4SD, Mean+5SD, Mean+6SD, Otsu's, and visual delineation methods were compared against the Mean+5SD LGE measurements, and their relative diagnostic performance was evaluated.

## Results

Relative to LGE images, mean infarct size and transmurality measured from native T_1_ maps were significantly over-estimated by Mean+2SD, Mean+3SD, and Mean+4SD techniques (p<0.001, for all cases). Mean+6SD criterion and visual delineation significantly underestimated infarct size (p<0.001 for both cases) and transmurality (p=0.01 for Mean+6SD; p<0.001 for visual) on native T_1_ maps. Otsu's technique showed no difference for measuring infarct size on native T_1_ maps compared to LGE images (p=0.27), but it over-estimated the infarct transmurality (p<0.001). Mean+5SD criterion showed no difference for measuring either infarct size (p=0.61) or transmurality (p=0.81) on T_1_ maps relative to LGE images. Mean CNR of LGE images was nearly 4-fold higher than that of native T_1_ maps (p<0.001). Mean+5SD criterion for detecting chronic MIs on native T_1_ maps at 3T showed the strongest diagnostic performance (area-under-curve=0.99, p<0.001), while visual delineation showed the weakest diagnostic performance (area-under-curve=0.84, p<0.001).

## Conclusions

Threshold-based analysis using Mean+5SD criterion can accurately and precisely estimate the size, location and transmurality of chronic MIs on native T_1_ maps as reliably as LGE at 3T.

## Funding

American Heart Association (13PRE17210049) and National Heart, Lung, And Blood Institute (HL091989).

**Figure 1:**
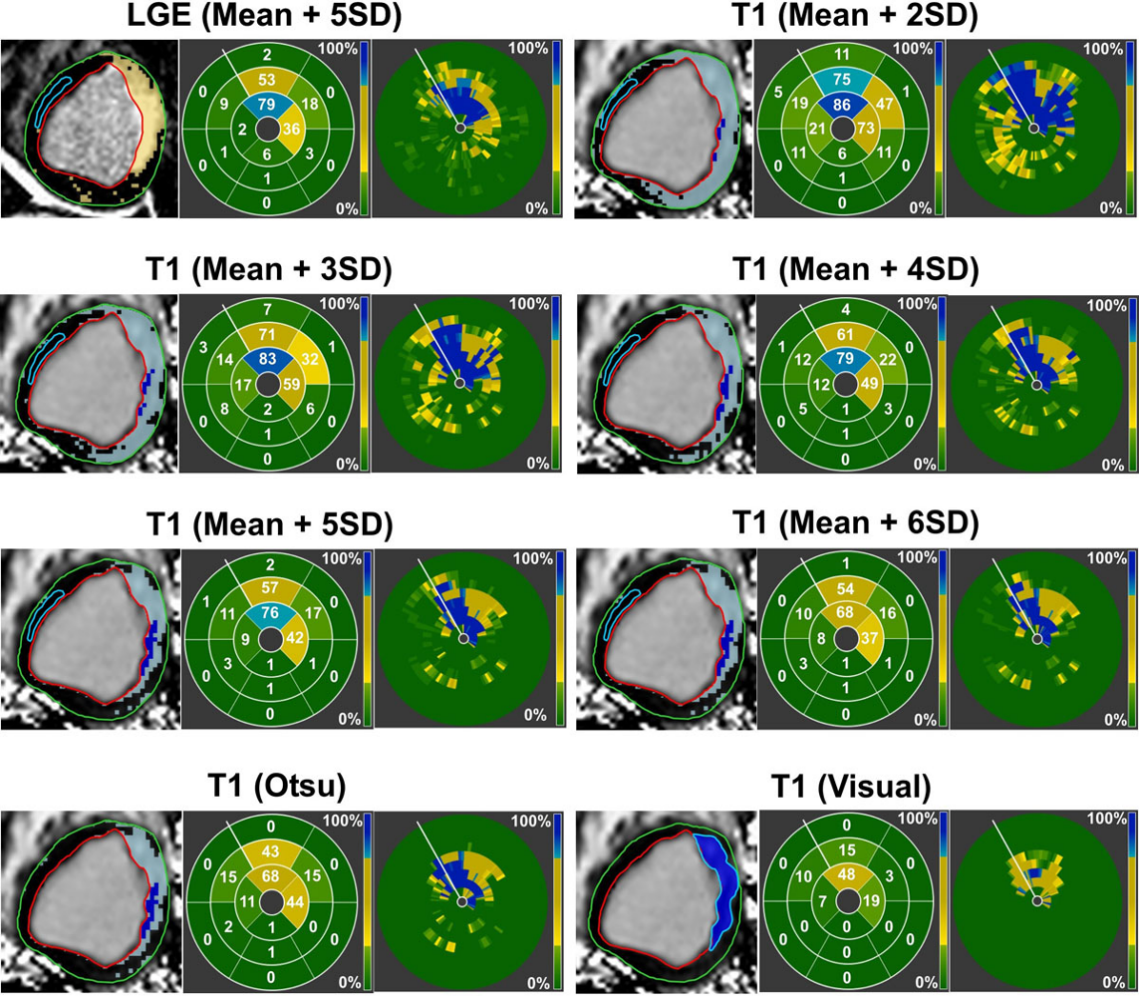
**Infarcted myocardium (highlighted pixels) detected using the Mean+5SD criterion on LGE images, and different thresholding criteria and visual delineation techniques on T**_**1**_ **maps are shown.** Relative to LGE images, significant over-estimation in the spatial extent, AHA-segmental infarct size, and transmurality could be observed with Mean+2SD and Mean+3SD techniques on T_1_ maps. Native T_1_ maps showed closer agreement to LGE images when Mean+4SD, Mean+5SD, Mean+6SD, and Otsu's techniques were used. Visual delineation significantly under-estimated the spatial extent and transmurality of the infarction on T_1_ maps.

**Figure 2:**
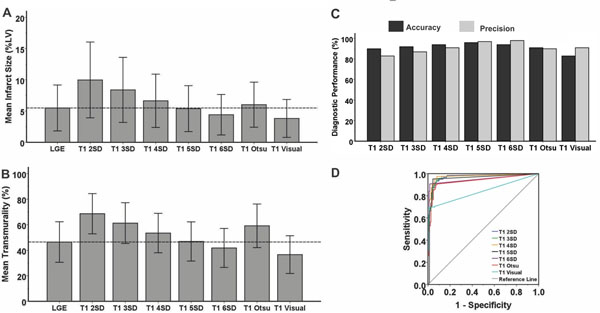
**Bar plots compare the mean infarct size (A) and transmurality (B) measured using different thresholding and visual delineation techniques on native T**_**1**_ **maps to those measured on LGE images using Mean+5SD criterion.** Bar plots comparing the accuracy and precision of different thresholding and visual delineation techniques to detect chronic MI are also shown (C). Of all the techniques used for infarct characterization on T_1_ maps, Mean+5SD criterion showed the best agreement to LGE images. ROC analysis (D) showed the strongest diagnostic performance for Mean+5SD technique, and the weakest diagnostic performance for visual delineation.

